# Highly efficient RNA-guided base editing in rabbit

**DOI:** 10.1038/s41467-018-05232-2

**Published:** 2018-07-13

**Authors:** Zhiquan Liu, Mao Chen, Siyu Chen, Jichao Deng, Yuning Song, Liangxue Lai, Zhanjun Li

**Affiliations:** 1 Jilin Provincial Key Laboratory of Animal Embryo Engineering, Institute of Zoonosis, Jilin University, Changchun, 130062 China; 2 Key Laboratory of Regenerative Biology, Guangzhou Institutes of Biomedicine and Health, Chinese Academy of Sciences, Guangzhou, Guangdong, 510530 China

## Abstract

Cytidine base editors (CBEs) and adenine base editors (ABEs), composed of a cytidine deaminase or an evolved adenine deaminase fused to Cas9 nickase, enable the conversion of C·G to T·A or A·T to G·C base pair in organisms, respectively. Here, we show that BE3 and ABE7.10 systems can achieve a targeted mutation efficiency of 53–88% and 44–100%, respectively, in both blastocysts and Founder (F0) rabbits. Meanwhile, this strategy can be used to precisely mimic human pathologies by efficiently inducing nonsense or missense mutations as well as RNA mis-splicing in rabbit. In addition, the reduced frequencies of indels with higher product purity are also determined in rabbit blastocysts by BE4-Gam, which is an updated version of the BE3 system. Collectively, this work provides a simple and efficient method for targeted point mutations and generation of disease models in rabbit.

## Abstract (web summary)

Base editors can make targeted changes without inducing a double-stranded break. Here, the authors apply the BE3 and ABE7.10 systems to rabbit to create highly efficient targeted base substitutions and various mutation types, and show reduced frequency of undesired by-products with the updated BE4-Gam system.

## Introduction

The vast majority of human genetic diseases arise from point mutations rather than insertions and deletions (indels), which has been verified through the explosive growth of human genomic data. Although the CRISPR/Cas9 system has been widely used to facilitate genome editing in a variety of organisms, including rabbits^1,2^, it would induce random indels through error-prone non-homologous end-joining (NHEJ) rather than the error-free homology-directed repair (HDR)^3^. As a result, indels are obtained much more frequently at target sites than single-nucleotide substitutions.

Recently, a programmable cytidine deaminase or adenine deaminase built on the CRISPR/Cas9 platform has been shown to achieve targeted C-to-T^4–6^ (BE3) or A-to-G^7^ (ABE7.10) conversion in living cells without generating DSBs or relying on template-donor DNA. To date, the BE3 system has undergone various improvement to increase the utility and applicability of the base editing capability^8–10^ and has been applied to induce single-nucleotide modifications in various plants and animals^11–14^. Similarly, ABE7.10 has also been reported to work in rice^15,16^ and mice^17^. However, both systems have not been assessed for efficacy and feasibility in rabbit.

In the present study, the BE3 and ABE7.10 systems were used to create targeted base substitutions in rabbit. The results provide a prospective application for the generation of rabbit models which could precisely mimic human genetic diseases. Furthermore, this is the first report, to our knowledge, demonstrating the reduction of frequencies of indels and improvement of product purity of the new BE version (BE4-Gam) in blastocysts, compared with BE3. Overall, we firstly demonstrate that base editors (both CBEs and ABEs) provide a simple and highly efficient method for inducing single-nucleotide substitution in rabbit.

## Results

### BE3 can induce C-to-T base conversion in rabbit blastocysts

**Fig. 1:**
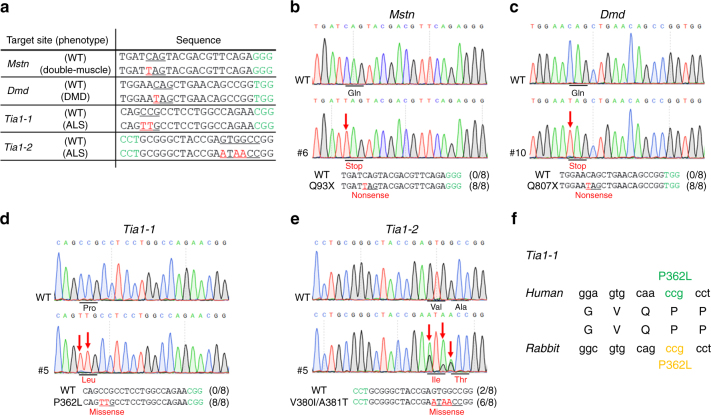
The BE3 system mediates efficient C-to-T base editing in rabbit blastocysts. **a** Target-site sequences within the *Mstn*, *Dmd*, *Tia1-1*, and *Tia1-2* loci. Target sequence (black), PAM region (green), target sites (red), and mutant amino acid (underlined). WT wild-type. **b**–**e** Representative sequencing chromatograms at the *Mstn*, *Dmd*, *Tia1-1*, and *Tia1-2* targets of WT and edited rabbit blastocysts. Target base editing (red arrows), target sequence (black), PAM region (green), target sites (red), mutant amino acid (underlined) and amino acid mutation type are indicated. The relevant codon identities at the target site are presented beneath the DNA sequence. **f** Diagrammatic representation of the mutations of *Tia1-1* associated with ALS and FTD in humans

To explore whether the BE3 system can catalyze site-specific base conversion in the rabbit genome in vivo, four target loci from three genes (*Mstn*, *Dmd*, and *Tia1*) were selected (Fig. 1a). Base editing was conducted in rabbit zygotes by microinjection of BE3-encoding mRNA and single guide RNAs (sgRNAs). Sanger sequencing and T–A cloning were performed to verify whether the targeted point mutations were successfully incorporated in each of the target site (Figs. 1b, c, d, e and Supplementary Fig. 1). It was observed that the mutation efficiency ranged from 75% to 87%, with an average successful target mutation rate of up to 70% (Table 1). Consistent with previous reports that BE3 system can still induce proximal off-targets, indels, or non-C-to-T conversions^4,13,18^, these undesired mutations were also observed in mutant blastocysts, albeit at low-average frequencies of 19%, 8%, or 3% (Table 1).

**Table 1:** Summary of embryo development and base editing rate using BE3 system

				Mutant ratio (%)				
Target gene	No. of zygotes	No. of 2-cell (%)^a^	No. of blastocysts (%)^a^	No. of mutants^b^	No. of targeted mutants^b^	No. of proximal off-targets^b^	No. of indels^b^	No. of non-C-to-T^b^
*Mstn*	31	29(94)	23(74)	20(87)	20(87)	4(17)	0(0)	0(0)
*Dmd*	23	19(83)	15(65)	12(80)	8(53)	1(7)	3(20)	1(7)
*Tia1-1*	25	22(88)	18(72)	15(83)	15(83)	8(44)	0(0)	0(0)
*Tia1-2*	22	20(91)	16(72)	12(75)	9(56)	1(6)	2(13)	1(6)
Average		(89.0 ± 4.6)	(70.7 ± 3.9)	(81.2 ± 5.0)	(69.7 ± 17.7)	(18.5 ± 17.7)	(8.2 ± 9.9)	(3.2 ± 3.7)

^a^Calculated from the number of zygotes

^b^Calculated from the number of blastocysts

Within these loci, missense mutations p.P362L and p.A381T in *Tia1* were recently implicated in the development of Amyotrophic Lateral Sclerosis (ALS) and Frontotemporal Dementia (FTD)^19^. The C–T conversion in *Tia1-1* induced the desired p.P362L amino acid change, precisely mimicking the p.P362L mutation observed in humans (Fig. 1f). These results indicate that BE3 system is simple and efficient in rabbit embryos, suggesting the potential of this system to improve economical traits and develop animal models for human genetic diseases in rabbits.

### Base conversion at *Mstn* to generate double-muscled rabbits

**Fig. 2:**
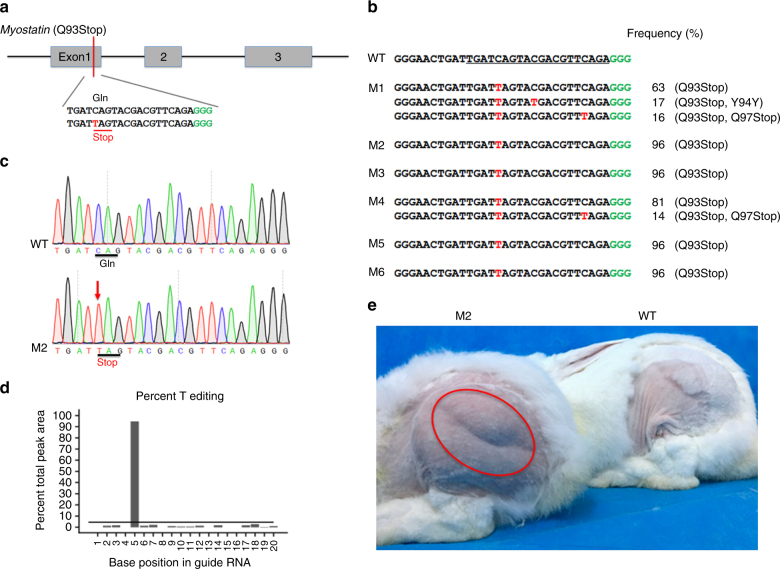
Generation of double-muscled rabbits using BE3 system. **a** The target sequence at the *Mstn* locus. The PAM-target and sgRNA-target sequences are shown in green and black, respectively. The substituted bases are marked in red. **b** Alignments of mutant sequences from targeted deep sequencing. The targeted sequence is underlined. PAM site and substitutions are shown in green and red, respectively. The column on the right indicates frequencies of mutant alleles. WT wild-type. **c** Sanger sequencing chromatograms of DNA from WT and *Mstn* mutant rabbits (M2). The red arrow indicates the substituted nucleotide. Relevant codon identities at the target site are presented beneath the DNA sequence. **d** The predicted editing bar plot based on Sanger sequencing chromatograms from M2 by EditR. **e** *Mstn* mutant rabbits exhibited a double-muscle phenotype (red circle, M2) at 3 months

*Mstn* is a member of the transforming growth factor beta (TGF-β) superfamily, which acts as a negative regulator of muscle growth. A double-muscled phenotype, with the characteristics of increased muscle mass, has been reported in *Mstn*-knockout (KO) sheep^20^, pigs^21^, dogs^22^, and rabbits^23^. Here, a single C-to-T conversion was designed to generate a premature stop codon (p.Gln93stop) in exon 1 of rabbit *Mstn* (Fig. 2a). It was expected that this mutation would inactivate the gene by directly converting normal coded codons into STOP codons^24^. Of the seven rabbits, this mutation was attempted in six (86%) of them who carried mutations at the target site (Fig. 2b and Table 2). Notably, targeted deep sequencing showed that all six mutant rabbits were homozygous for a nonsense mutation, as was expected. In particular, four (M2, M3, M5, and M6) out of six rabbits also showed a precise-targeted single C-to-T conversion in the absence of other unwanted mutations (Figs. 2b, c and Supplementary Fig. 2a). The results from EditR, a novel base editing quantification software^25^, also confirmed the efficient C-to-T conversion, which was consistent with the deep-sequencing data (Figs. 2d, b). In addition, no off-target mutations were detectably induced at potential off-target sites (POTs) in *Mstn* mutant rabbits (Supplementary Fig. 3a). Furthermore, the expected double-muscle phenotype was observed in mutants at 3 months of age, relative to their WT counterparts (Fig. 2e). Overall, these data suggested that BE3 system can induce highly accurate and efficient single base editing in Founder rabbits. This technology possesses great prospects for the improvement of economically desirable traits in animal production.

**Table 2:** Generation of base conversions in Founder rabbits using BE3 and ABE7.10 system

			Mutant ratio (%)					
System	Target gene	No.of offspring	No. of mutants	No. of target mutants	No. of homozygous target mutants	No. of proximal off-targets	No. of non-C-to-T/ A-to-G	No. of indels
BE3	*Mstn*	7	6(86)	6(86)	4(57)	2(29)	0(0)	0(0)
BE3	*Tyr*	7	6(86)	6(86)	0(0)	6(86)	1(14)	1(14)
BE3	*Lmna*	8	7(88)	7(88)	0(0)	7(88)	0(0)	0(0)
ABE7.10	*Dmd*	7	6(86)	6(86)	0(0)	1(14)	0(0)	0(0)
Average			(86.5 ± 1.0)	(86.5 ± 1.0)	(14.2 ± 28.5)	(54.2 ± 38.3)	(3.5 ± 7.0)	(3.5 ± 7.0)

### Base conversion at *Tyr* to recapitulate human albinism

**Fig. 3:**
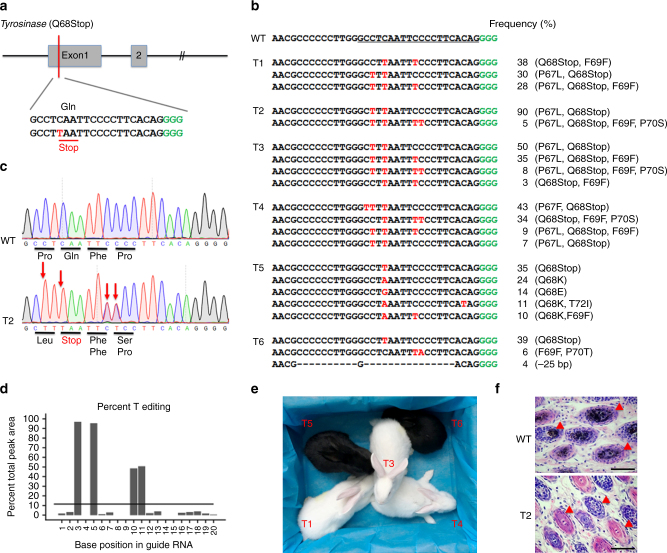
Generation of an albinism rabbit model using BE3 system. **a** The target sequence at the *Tyr* locus. The PAM- and sgRNA-target sequences are shown in green and black, respectively. The substituted bases are marked in red. **b** Alignments of mutant sequences from targeted deep sequencing. The targeted sequence is underlined. PAM site and substitutions are shown in green and red, respectively. The column on the right indicates frequencies of mutant alleles. WT wild-type. **c** Sanger sequencing chromatograms from WT and *Tyr* mutant rabbits (T2). The red arrow indicates the substituted nucleotide. The relevant codon identities at the target site are presented under the DNA sequence. **d** The predicted editing bar plot based on Sanger sequencing chromatograms from T2 by EditR. **e** *Tyr* mutant rabbits with homozygous nonsense mutations exhibited a systemic albino phenotype (T1, T3, and T4). **f** H&E staining of skin from WT (T7) and *Tyr* mutant (T2) rabbits. The red triangle highlights the melanin in the basal layer of the epidermis. Scale bars: 50 μm

It is widely accepted that the *Tyr* gene is the major causal gene for human ocular albinism (OA) and oculocutaneous albinism (OCA)^26,27^. As with other conditions, a single C-to-T conversion is expected to yield a premature stop codon (p.Gln68stop) in the gRNA-target region. Seven pups were obtained for the mutagenesis study (Fig. 3a). Targeted deep sequencing showed that four newborn rabbits (T1, T2, T3, and T4) were homozygous for a nonsense mutation at the target site (Fig. 3b). Meanwhile, the additional cytosines (Cs) proximal to the targeted base were also converted to thymines (Fig. 3c, d and Supplementary Fig. 2b). This observation is consistent with previous reports in living cells^4^. Moreover, C-to-A and C-to-G conversions were also observed in one mutant rabbit (T5), while another one (T6) had a mild 25-base-pair (bp) deletion rather than point mutation. Similarly, no off-target mutations were detectably induced at POTs in *Tyr* mutant rabbits (Supplementary Fig. 3b). Four out of seven pups (57%) showed an albino phenotype systemically. The other two mutants (T5 and T6) remained black in color, which was consistent with their mutant genotype (Fig. 3e). In addition, histological H&E staining revealed the absence of melanin in hair follicles of T2, but not in the WT littermate (Fig. 3f). These results showed efficient base substitution at the *Tyr* locus by the BE3 system and the potential of it to carry out loss-of-functional strategy as an alternative to gene KO studies by WT Cas9.

### Base conversion at *Lmna* to mimic the HGPS mutation

**Fig. 4:**
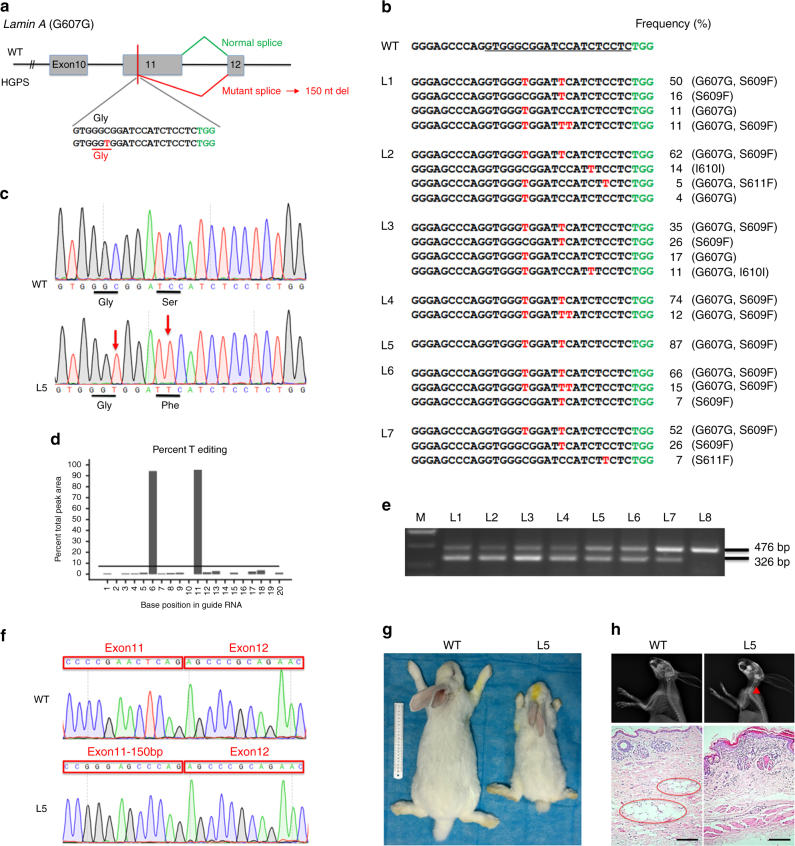
Generation of a HGPS rabbit model using BE3 system. **a** The target sequence at the *Lmna* locus. The PAM- and sgRNA-target sequences are shown in green and black, respectively. The substituted bases are marked in red. Green and red lines indicate the normal and mutant mRNA splice forms, respectively. Mutant mRNA splicing results in a hypothetical 150-nucleotide deletion. **b** Alignments of mutant sequences from targeted deep sequencing. The targeted sequence is underlined. PAM site and substitutions are shown in green and red, respectively. The right column indicates the frequencies of the observed mutant alleles. WT wild-type. **c** Sanger sequencing chromatograms from WT and *Lmna* mutant rabbits (L5). The red arrow indicates the substituted nucleotide. Relevant codon identities at the target site are represented below the DNA sequence. **d** The predicted editing bar plot based on Sanger sequencing chromatograms from L5 by EditR. **e** Demonstration of the abnormal splice product using RT-PCR, showing a spliced product of 326 bp in mutant rabbits (L1 to L7) due to activation of the cryptic splice site. **f** Sanger sequencing chromatograms of the abnormal RT-PCR product confirmed the deletion of 150 nucleotides within exon 11 in the mutant rabbit (L5). **g** Photographs of WT (L8, 2.56 kg) and *Lmna* mutant (L5, 0.93 kg) rabbits at 3 months. **h** Radiograph and H&E staining of the skin from a 3-month-old *Lmna* mutant rabbit (L5) compared with a WT littermate (L8). The red triangle highlights the curvature of the spine. Red circles indicate subcutaneous adipose tissue. Scale bars: 200 μm

Hutchinson–Gilford progeria syndrome (HGPS) is a rare genetic disorder that causes premature, rapid aging shortly after birth^28^. For the vast majority of cases, a de novo point mutation (p.G608G) in the *Lmna* gene results in the development of HGPS. This synonymous mutation creates a cryptic splice donor site that produces a mutant lamin A protein, termed “progerin”, which carries a 50-aa deletion near the C-terminus^29^. Here, a sgRNA was designed to replace the WT rabbit *Lmna* gene with a mutant allele carrying the c.1821C > T;p.G607G mutation, which is equivalent to the HGPS c.1824C > T;p.G608G mutation in the human *Lmna* gene (Fig. 4a). In previous reports, BE3 system showed a striking preference for TC, and the editing efficiency of GC, in general, is quite low in living cells^4,24^. However, in the present study, targeted point mutations were observed in seven out of eight (88%) rabbits at the target site (Fig. 4b, Supplementary Fig. 2c and Table 2). Similarly, proximal off-targets were still observed in mutant rabbits (Fig. 4b). Notably, two pup (L4, L5) harbored only the mutant allele, which induced p.G607G and p.S609F mutations of rabbit *Lmna* gene (Figs. 4b, c, d). Taken together, these results show an efficient C-to-T conversion at the specified locus, with a G motif on the immediate 5’ end of the targeted cytosine. These data suggest that the BE3 system may exhibit better site adaptability in rabbits. Moreover, we could not detect off-target mutations at POTs in *Lmna* mutant rabbits (Supplementary Fig. 3c). In order to determine whether the mutation resulted in RNA mis-splicing in exon 11, as is commonly observed in cases of human HGPS, RT-PCR analysis showed an additional smaller product (Fig. 4e and Supplementary Fig. 4), and a 150-nucleotide deletion was observed in mutant rabbits by Sanger sequencing (Fig. 4f and Supplementary Fig. 5). This observation is completely consistent with RNA mis-splicing observed in human HGPS patients. Furthermore, the typical phenotype of growth retardation, short stature (Fig. 4g), bone abnormalities, and loss of subcutaneous fat (Fig. 4h) were also observed in the *Lmna* mutant rabbits^28,29^. Thus, this rabbit model precisely mimics the human HGPS mutation and disease, underscoring the great potential of the BE3 system to efficiently generate point mutation disease models in rabbits.

### Higher product purity using BE4-Gam system

Although the BE3 system could efficiently induce base conversion in rabbit, it still induced the unwanted indels and non-C-to-T conversions in the *Dmd* and *the Tia1-2* genes. Indel mutations were observed in three out of 15 (20%) or two out of 16 (13%) blastocysts at the target site in the *Dmd* or the *Tia1-2* gene, respectively (Table 1 and Supplementary Figs. 1b, d). Meanwhile, non-C-to-T conversions were observed in one out of 15 (7%) or one out of 16 (6%) blastocysts in the above gene, respectively (Table 1 and Supplementary Figs. 1b, d). And these untargeted mutations would cause unwanted frameshift or missense mutations which were deleterious and imprecise to build models and treat genetic diseases. Recently, the BE4-Gam system, with Gam protein from bacteriophage Mu fused to the N-terminus, and appending a second copy of UGI to the C-terminus of BE3, has been reported (Supplementary Fig. 6). The reduced frequencies of indels and higher product purity were determined in human cells compared to BE3^10^. However, the efficacy of this system has not been reported in mammalian embryos.

Thus, to determine if the BE4-Gam system could help to overcome these shortcomings of BE3, the loci of *Dmd* and *Tia1-2* were performed to comparatively evaluate both systems (BE3 and BE4-Gam) in rabbit embryos. As shown in Table 3, significant increase in the targeted mutation efficiency, reduction in the frequency of indels, and not observed non-C-to-T conversions at both loci were determined in the BE4-Gam system. Meanwhile, there is no significantly different embryonic development rate and mutation efficiency between BE3 and BE4-Gam (Table 3, Supplementary Figs. 7, 8), suggesting that BE4-Gam is a reliable base editing tool for use in rabbit embryos. Taken together, these results indicate that BE4-Gam offers higher product purities and lower indel rates at both loci tested, which will serve as a significant advancement in C-to-T conversion, as well as in the utility and applicability of base editing.

**Table 3:** BE4-Gam system improves product purity in rabbit blastocysts

				Mutant ratio (%)				
Gene	System	No. of 2-cell(%)^a^	No. of blastocysts(%)^a^	No. of mutants^b^	No. of target mutants^b^	No. of proximal off-targets^b^	No. of indels^b^	No. of non-C-to-T^b^
*Dmd*	BE3	(89.3 ± 1.1)^c^	(79.3 ± 5.1)^c^	(82.3 ± 4.5)^c^	(52.3 ± 6.8)^c^	(8.6 ± 2.8)^c^	(21.6 ± 2.0)^c^	(8.6 ± 2.8)^c^
	BE4-Gam	(89.6 ± 4.5)^c^	(79.3 ± 1.1)^c^	(85.0 ± 3.6)^c^	(80.3 ± 1.1)^d^	(4.0 ± 3.4)^c^	(4.3 ± 3.7)^d^	(0.0 ± 0.0)^d^
*Tia1-2*	BE3	(83.6 ± 3.2)^c^	(75.6 ± 1.1)^c^	(80.6 ± 1.1)^c^	(46.3 ± 0.5)^c^	(21.3 ± 2.3)^c^	(25.3 ± 4.7)^c^	(8.6 ± 3.7)^c^
	BE4-Gam	(85.3 ± 5.0)^c^	(77.6 ± 6.4)^c^	(79.0 ± 2.6)^c^	(75.0 ± 5.2)^d^	(21.3 ± 8.1)^c^	(4.3 ± 3.7)^d^	(0.0 ± 0.0)^d^

^a^Calculated from the number of zygotes

^b^Calculated from the number of blastocysts

^c,d^Values with different superscripts within the same column denote significant difference

### ABE7.10 induces A-to-G base conversion in rabbit blastocysts

**Fig. 5:**
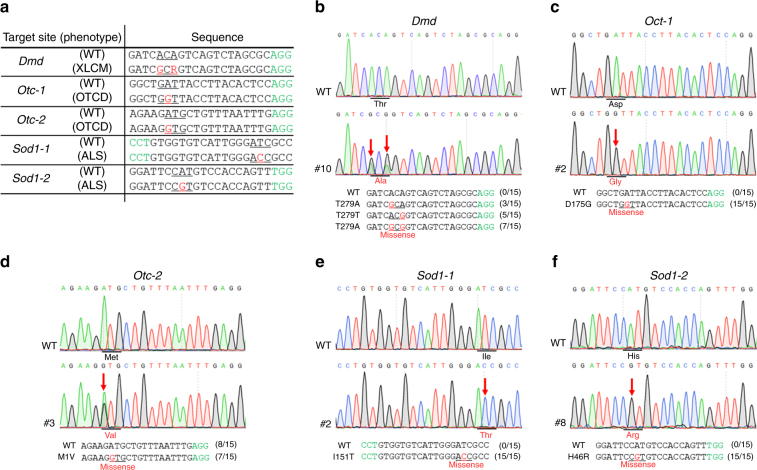
ABE7.10 induces the efficient A-to-G base editing in rabbit blastocysts. **a** Target site sequences within the *Dmd*, *Otc-1*, *Otc-2*, *Sod1-1,* and *Sod1-2* loci. Target sequence (black), PAM region (green), target sites (red, R = A/G), and mutant amino acid (underlined). WT wild-type. **b–f** Representative sequencing chromatograms at *Dmd*, *Otc-1*, *Otc-2*, *Sod1-1*, and *Sod1-2* targets of WT and edited rabbit blastocysts. Target base editing (red arrows), target sequence (black), PAM region (green), target sites (red), mutant amino acid (underlined), and amino acid mutation type are indicated. The relevant codon identities at the target site are presented beneath the DNA sequence

Despite the high efficiency of BE3 system-mediated C-to-T conversion, additional base editing tools such as recently reported ABE system are needed for increasing the versatility. To explore whether the ABE7.10 system can mediate the site-specific A·T to G·C conversion in the rabbit genome in vivo, five target loci from three genes (*Dmd, Otc*, and *Sod1*) were selected (Fig. 5a). These target loci with A·T to G·C conversion in *Dmd* (T279A), *Otc* (D175G, M1V), and *Sod1* (I151T, H46R) have been reported to cause X-linked dilated cardiomyopathy (XLCM)^30^, ornithine transcarbamylase deficiency (OTCD)^31^, and amyotrophic lateral sclerosis (ALS)^32,33^, respectively. Targeted point mutations were observed in all target loci, with high efficiency from 44.4% (4/9, *Sod1-2*) to 100.0% (10/10, *Sod1-1*) (Figs. 5b, c, d, e, Table 4 and Supplementary Fig. 9). Meanwhile, allelic frequencies of up to 100% were observed in *Otc-1* (#2, #6, #9), *Sod1-1* (#2), *and Sod1-2* (#8) loci, with no evidence of mosaicism (Figs. 5c, e, f and Supplementary Fig. 9). Notably, targeted point mutation in *Otc-1* were observed in eight out of nine (88.9%) blastocysts, and homozygous mutation efficiency of up to 44.4% (4/9) was observed (Table 4 and Supplementary Fig. 9b). In addition, we found that the adenines at positions 5–8 of the protospacer could be efficiently converted to guanines, and neither indels nor any other nucleotide changes were observed at the target sites, which is consistent with the result of Liu’s group in mammalian cells^7^. Furthermore, no off-target mutations were detectable at POTs in these loci of mutant blastocysts (Supplementary Fig. 10). Overall, these results suggested that the ABE7.10 system is highly efficient and precise in the conversion of the A·T to G·C base pair in rabbit blastocysts.

**Table 4:** Summary of embryo development and base editing rate using ABE7.10 system

				Mutant ratio (%)				
Target gene	No. of zygotes	No. of 2-cell(%)^a^	No. of blastocysts(%)^a^	No. of mutants^b^	No. of target mutants^b^	No. of proximal off-targets^b^	No. of indels^b^	No. of non-A-to-G^b^
*Dmd*	18	15(83)	11(61)	9(82)	9(82)	0(0)	0(0)	0(0)
*Otc-1*	15	13(87)	9(60)	8(89)	8(89)	0(0)	0(0)	0(0)
*Otc-2*	14	11(79)	9(64)	6(67)	6(67)	2(22)	0(0)	0(0)
*Sod1-1*	15	12(80)	10(67)	10(100)	10(100)	0(0)	0(0)	0(0)
*Sod1-2*	16	12(75)	9(56)	4(44)	4(44)	1(11)	0(0)	0(0)
Average		(80.8 ± 4.4)	(61.6 ± 4.1)	(76.4 ± 21.7)	(76.4 ± 21.7)	(6.6 ± 9.8)	(0.0 ± 0.0)	(0.0 ± 0.0)

^a^Calculated from the number of zygotes

^b^Calculated from the number of blastocysts

### Base conversion at *Dmd* to mimic the XLCM mutation

**Fig. 6:**
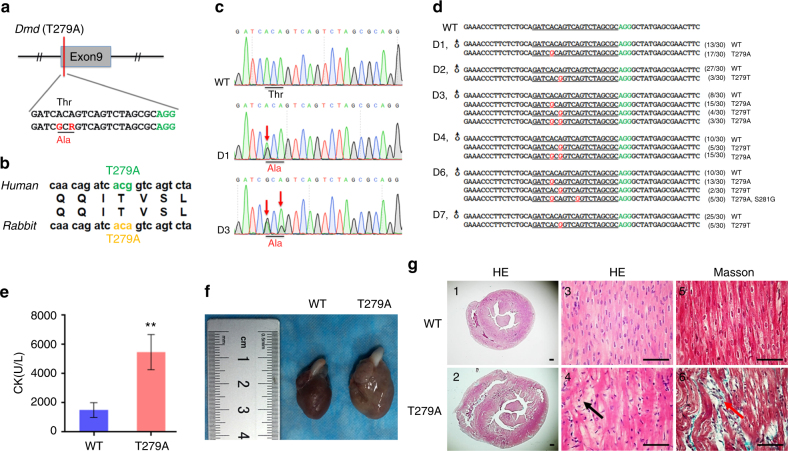
Generation of an XLCM rabbit model using the ABE7.10 system. **a** The target sequence within the *Dmd* locus. Target sequence (black), PAM region (green), and substituted nucleotide (red, R = A/G). **b** Diagrammatic representation of mutations associated with XLCM in humans and rabbits. **c** Representative Sanger sequencing chromatograms of DNA from WT and mutant rabbits (D1 and D3). Red arrow indicates the substituted nucleotide. The relevant codon identities at the target site are presented beneath the DNA sequence. WT wild-type. **d** PCR amplicons of the target site from genomic DNA of mutant rabbits were subcloned into the pGM-T vector and sequenced. The number of clones for each sequence pattern is indicated. Target sequence (underlined), PAM region (green), and substituted nucleotide (red). **e** Serum CK levels of WT and T279A mutant rabbits (*n* = 4). **p < 0.01, Student’s *t* test. **f** Representative gross morphology of whole hearts from WT and T279A mutant rabbits at 1 month of age. **g** Transverse cross sections at mid-ventricle level of whole hearts. Corresponding H&E-stained and Masson’s trichrome-stained longitudinal sections of cardiac muscle showed myocyte disarray (black arrow) and fibrosis (red arrow). Scale bar in 1 and 2 represent 0.4 mm and scale bar in 3, 4, 5, and 6 represent 50 μm

A previous study has shown that p.T279A mutation in exon 9 of *Dmd* causes the cardio-specific phenotype of XLCM and the highly conserved coding sequence between humans and rabbits^30^ (Figs. 6a, b). Subsequently, we transplanted rabbit embryos into surrogate mothers after microinjection. The result showed that six out of eight (75%) rabbits carried mutations at the target site and four of them (50%) carried the desired missense mutation (Figs. 6c, d, Supplementary Fig. 11 and Table 2). As expected, ABE7.10 showed a precise base editing window at positions 5–7 of the protospacer in the F0 rabbits, except an extra A-G substitution at position 11 in the D6 rabbit (5/30), which is consistent with data from the embryo research (Fig. 5b). Similarly, no indels or other mutations were detected in F0 rabbits (Fig. 6d). No off-target mutations were detectable at POTs, suggesting that ABE7.10 is efficient and precise to introduce point mutations (Supplementary Fig. 12). Furthermore, T279A mutant rabbits showed significantly elevated serum creatine kinase (CK) and sudden death within 1 month (Fig. 6e). Dissection and histologic examination showed the  dilated hearts with myocyte disarray and fibrosis, while no obvious pathological change were determined in diaphragm muscle and gastrocnemius of the T279A mutant rabbits (Figs. 6f, g and Supplementary Fig. 13). Together, T279A mutant rabbits displayed typical clinical symptoms consistent with human XLCM rather than Duchenne muscular dystrophy (DMD), confirmed the specific correlation between p.T279A mutation and XLCM^30^.

## Discussion

To our best knowledge, this is the first report that highly efficient base conversion can be achieved in rabbit without the use of template DNA. Our results demonstrated that the BE3 and ABE7.10 system can induce site-specific, single-base substitutions with efficiency rates of 53–88% or 44–100% in the rabbit, respectively (Tables 1, 2 and 4). In addition, we demonstrated that the new BE version (BE4-Gam) significantly reduced frequencies of indels, non-C-to-T conversions, and significantly increased targeted mutation efficiency in rabbit blastocysts compared to its predecessor (Table 3). Furthermore, probing for off-target effects showed that both the CBEs and ABEs did not result in off-target effects in base-edited rabbits (Supplementary Figs. 3, 10, and 12). This finding is consistent with a previous report which demonstrated the high specificity of this system in humans and mice^13,17,34^. These results support that the BE3 and ABE7.10 systems are highly efficient and specific, and can be used as reliable tools for targeted base editing in rabbit.

The vast majority of human genetic diseases are caused by point mutations, with C:G to T:A and A:T to G:C mutations accounting for approximately half of all known pathogenic SNPs^7^. While most animal models are constructed by the knockout of a specific gene, it therefore does not precisely mimic diseases caused by point mutations in humans. In case of diseases caused by dominant mutations, simple gene knockouts cannot mimic the function gained by the mutation^12^. In this work, we describe the precise base editing of the *Tia1* gene to mimic the human missense mutations p.P362L and p.A381T (Figs. 1d, e and Table 1). In addition, the p.G607G mutation at the *Lmna* locus or the p.T279A mutation at the *Dmd* locus create clinical signs and symptoms associated with classical HGPS mutation (p.G608G) or XLCM mutation (p.T279A) in humans (Figs. 4a, 5a). Mutant rabbits produced here showed the typical phenotypes observed in HGPS or XLCM patients. These results suggested that BE3 and ABE7.10 systems are able to effectively build animal models precisely mimicking diseases caused by point mutations in humans.

In addition, we found that ABE7.10 showed precise targeted A-to-G conversion with few proximal off-targets (Tables 2 and 4), which is consistent with the previous report in mammalian cells^7^. However, proximal off-targets were still observed at target loci in relatively high proportion using BE3 system (Tables 1 and 2). This may be arised from the relatively wide editing window, and the high activity of APOBEC1 likely contributes to the deamination of multiple Cs per DNA binding event, consistent with the previous report from David Liu’s group^8^. It may be reduced through the optimization of rAPOBEC1 with a mutant deaminase domain such as YEE-BE3 version to effectively narrow the deamination window^8^. Moreover, a recent preprint showed an alternative strategy for reducing bystander mutations using a novel BE architecture with an engineered human APOBEC3A (eA3A) domain^35^. Thus, engineering cytidine deaminases may be an effective strategy to improve the precision of base editors in future.

Actually, the current mainstream approach to generate animals with desired base conversion is using CRISPR/Cas9 system with ssODN via HDR. But it is less effective compared to CBE system and ABE system, and will produce many undesired indel mutations, as per previous reports^36–38^. Recently, Easi-CRISPR using long ssDNAs has been reported as a high-efficient technology, and it may boost the development of knock-in methods. In comparison, base editors have the advantage that it does not require formation of double-stranded DNA breaks or provision of a donor DNA template, while larger sequences can be inserted via HDR-mediated knockin, notably loxP sequences. Overall, base editor systems will significantly benefit the animal disease models generation and gene therapy in pre-clinical study.

In summary, the BE3 and ABE7.10 systems are highly efficient at inducing base conversions in rabbits in a programmable manner, and the frequency of undesired by-products has been significantly reduced when BE3 system was upgraded to the BE4-Gam. It was also confirmed that both CBE and ABE systems can be used to create a variety of mutation types, including nonsense mutations, missense mutations, and RNA mis-splicing by single base editing. Taken together, the CBE and ABE systems provide a simple and efficient method through which rabbit models can be produced to precisely mimic mutations associated with disease conditions in humans.

## Methods

### Ethics statement

New Zealand white and Lianshan black rabbits were obtained from the Laboratory Animal Center of Jilin University (Changchun, China). All animal studies were conducted according to experimental practices and standards approved by the Animal Welfare and Research Ethics Committee at Jilin University.

### mRNA and gRNA preparation

pCMV-BE3, BE4-Gam, and ABE7.10 plasmids were obtained from Addgene (#73021, #100806 and #102919). The plasmid was linearized with Not I, and mRNA was synthesized using an in vitro RNA transcription kit (HiScribe™ T7 ARCA mRNA kit (with tailing), NEB). sgRNA oligos were annealed into pUC57-sgRNA expression vectors with T7 promoter. Then, sgRNAs were amplified and transcribed in vitro using the MAXIscript T7 kit (Ambion) and purified with miRNeasy Mini Kit (Qiagen) according to manufacturer’s instructions. sgRNA-oligo sequences used in this study were listed in Supplementary Table 1.

### Microinjection of rabbit zygotes

The protocol for microinjection of pronuclear-stage embryos has been described in detail in our published protocols^39^. Briefly, a mixture of BE3, BE4-Gam, or ABE7.10 mRNA (200 ng/ul), and sgRNA (50 ng/ul) was co-injected into the cytoplasm of pronuclear-stage zygotes.

### Single embryo PCR amplification and rabbit genotyping

Injected embryos were collected at the blastocyst stage. Genomic DNA was extracted with an embryo lysis buffer (1% NP40) at 56 °C for 60 min and 95 °C for 10 min in a BIO-RAD PCR Amplifier, and then subjected to Sanger sequencing. Genomic DNA was extracted from ear clips of newborn rabbits for PCR genotyping and subjected to Sanger sequencing, T-A cloning, or targeted deep sequencing. All primers for detection are listed in Supplementary Data 1.

### Targeted deep sequencing

Targeted sites were amplified from genomic DNA using Phusion polymerase (Thermo Fisher Scientific).The paired-end sequencing of PCR amplicons was performed by Sangon Biotech (Shanghai), using an Illumina MiSeq.

### Off-target assay

Ten potential off-target sites (POTs) for each sgRNA were predicted to analyze site-specific edits according to an online design tool (http://crispr.mit.edu/) and Cas-OFFinder (http://www.rgenome.net/cas-offinder/)^40^. The PCR products of the POTs were sequenced. All primers for off-target assay are listed in Supplementary Datas 2 and 3.

### Haematoxylin and eosin (H&E), and Masson’s trichrome staining

The protocol has been described in detail in our published protocols^41^. Briefly, tissues from WT and mutant rabbits were fixed in 4% paraformaldehyde for 48 h, embedded in paraffin wax, and then sectioned for slides. Slides were stained with H&E and Masson’s trichrome, and viewed under a Nikon ts100 microscope.

### X-ray detection

X-ray autoradiography pictures of whole-body skeletons and bones of interest were taken using the YEMA Radiography System with a digital camera attached (Varian, USA) on X-ray film (ROTANODE, Japan). Images were taken at 40 kV with 3 mAs exposure.

### Serum CK analysis

WT and *Dmd* mutant rabbits were anesthetized and blood was collected. Serum samples were obtained by precipitation and centrifugation. Serum CK level was measured using Roche/Hitachi cobas c 701 analyzer in KingMed Diagnostics (Changchun, Ji Lin).

### Statistical analysis

All data are expressed as mean ± SD, at least three individual determinations in all experiments. Data were analyzed with t-tests using Graphpad prism software 6.0. A probability of *p* < 0.05 was considered statistically significant. *p *<* 0.05, **p *<* 0.01, ***p *<* 0.001.

### Data availability

Sequencing data from this work has been deposited at the Sequence Read Archive under accession code SRP151162 Authors state that all data necessary for confirming the conclusions presented in the article are represented fully within the article or available from authors upon request.

## Electronic supplementary material

Supplementary Information

Description of Additional Supplementary Files

Supplementary Data 1

Supplementary Data 2

Supplementary Data 3
