# Supplementary material for: Highly efficient RNA-guided base editing in rabbit
Source: Nat Commun. 2018 Jul 13;9:2717. doi: 10.1038/s41467-018-05232-2 (PMC6045575; doi:10.1038/s41467-018-05232-2)
Supplement: Supplementary file 1 — Supplementary Information [file 41467_2018_5232_MOESM1_ESM.pdf]

Highly efficient RNA-guided base editing in rabbit

Liu et al

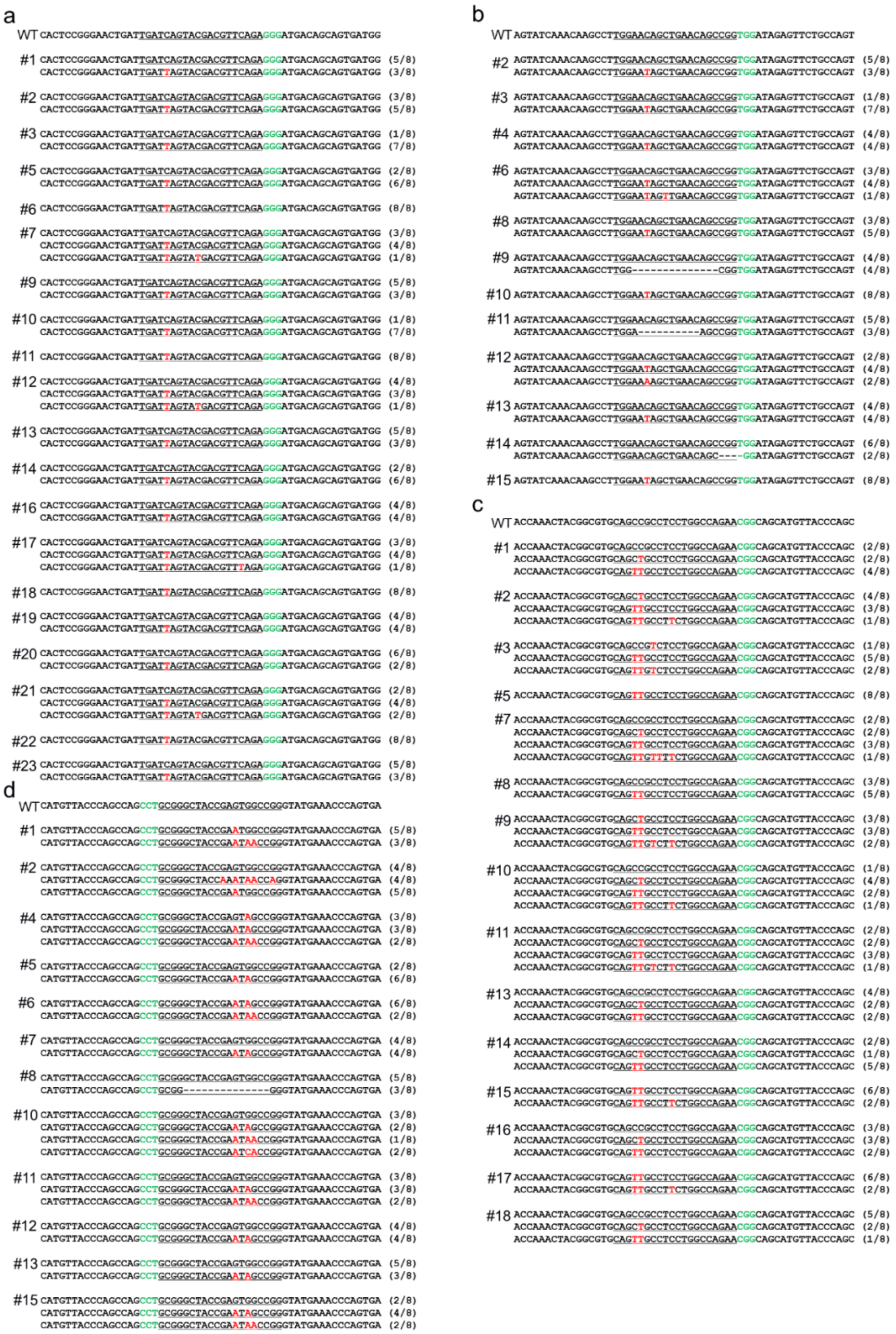

Supplementary Figure 1: **Targeted base editing in rabbit blastocysts using BE3 system.** (a-d): PCR amplicons of *Mstn* (a), *Dmd* (b), *Tia1-1* (c) and *Tia1-2* (d) loci from the genomic DNA of mutant blastocysts were subcloned into pGM-T vectors and sequenced, respectively. The number of clones for each sequence pattern is indicated. Target sequence (underlined), PAM region (green), and substituted nucleotide (red).

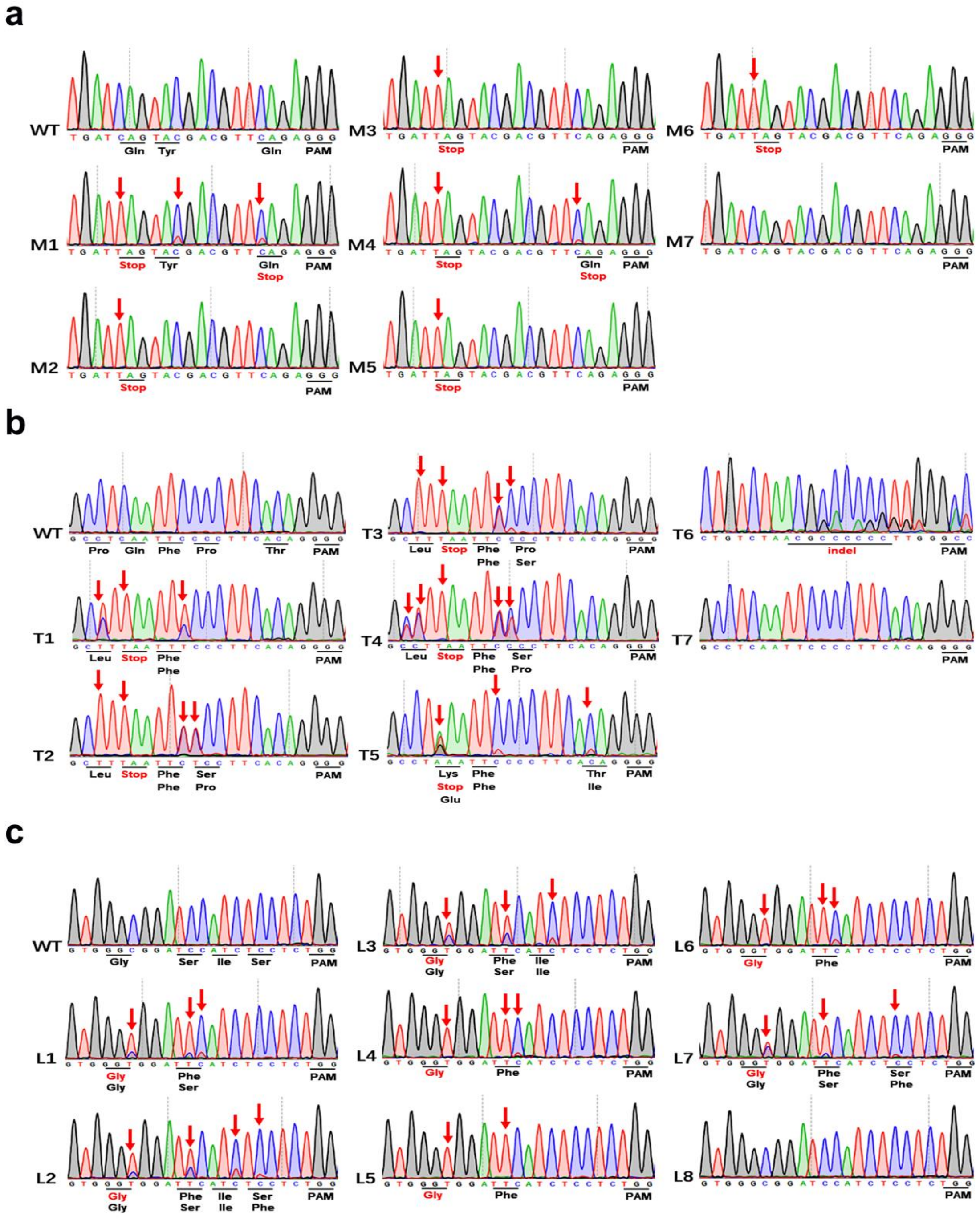

Supplementary Figure 2: **Sanger sequencing chromatograms of WT and mutant Founder rabbits.** (a-c)**:** Sanger sequencing chromatograms of genomic DNA from all *Mstn* (a)*, Tyr* (b)*, Lmna* (c) Founder rabbits, respectively. The red arrow indicates the substituted nucleotide. The relevant codon identities at the target site are shown under the DNA sequence.

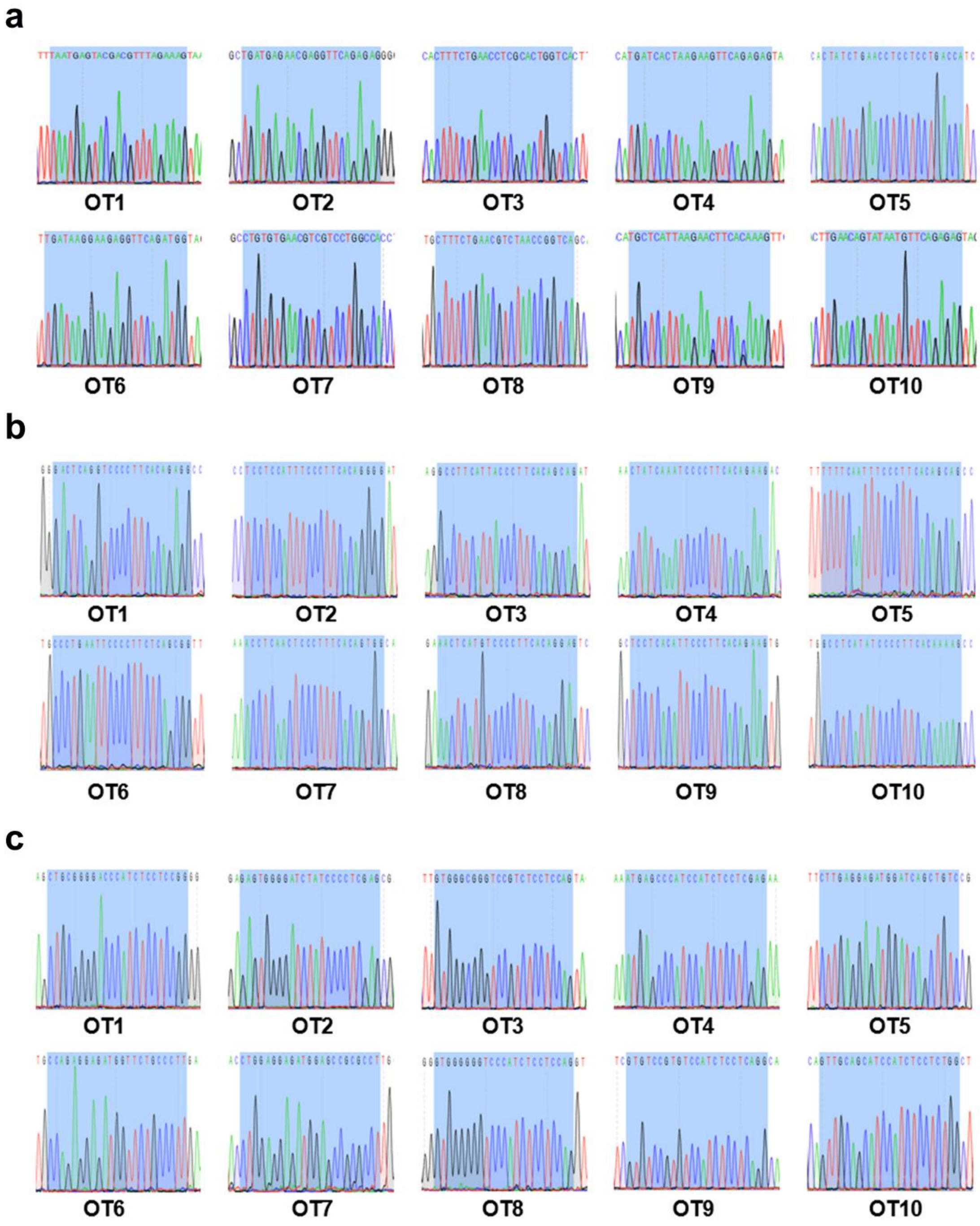

Supplementary Figure 3: **Off-target detection in the mutant Founder rabbits using BE3 system.** (a-c): Chromatogram sequence analysis of ten potential off-target sites (POTS) for sgRNA using PCR products in *Mstn* (a), *Tyr* (b), *Lmna* (c) mutant Founder rabbits, respectively. 20 bp of the POTS and the PAM are represented in shadow.

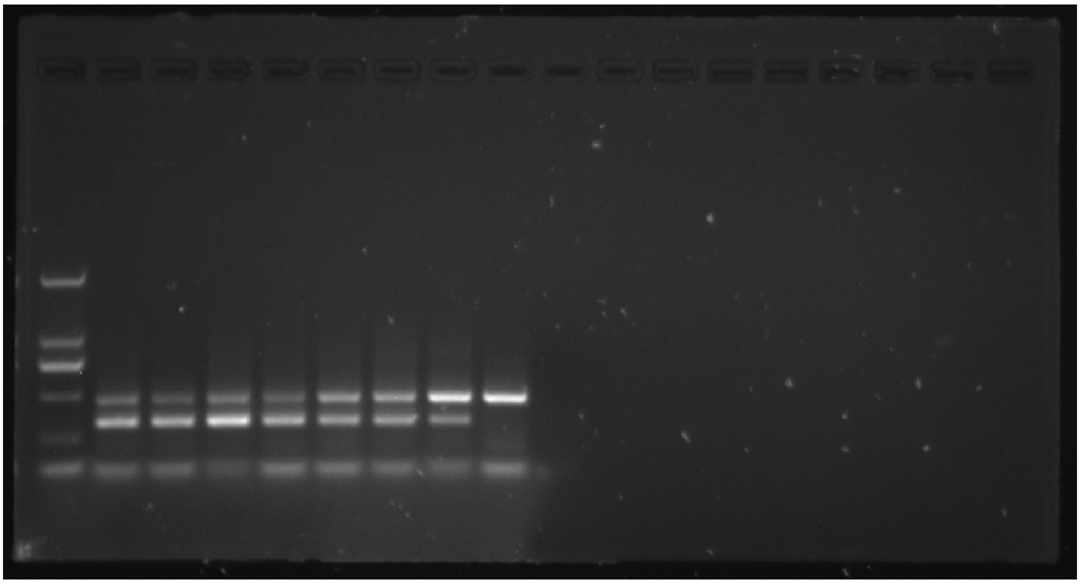

Supplementary Figure 4: **Uncropped gels of Fig. 4e.** Demonstration of the abnormal splice product using RT-PCR, showing a spliced product of 326bp in mutant rabbits (L1 to L7) due to activation of the cryptic splice site.

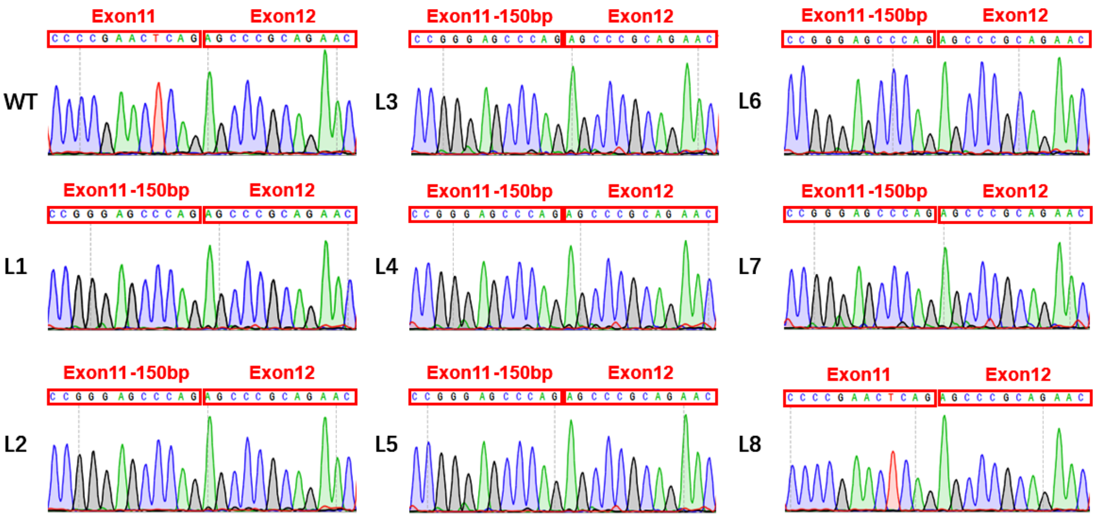

Supplementary Figure 5: **Sanger sequencing chromatograms of RT-PCR from WT and *Lmna* mutant Founder rabbits.** Sanger sequencing chromatograms of abnormal product from RT-PCR confirmed the missing of 150 nucleotides within exon 11 in the mutant rabbits.

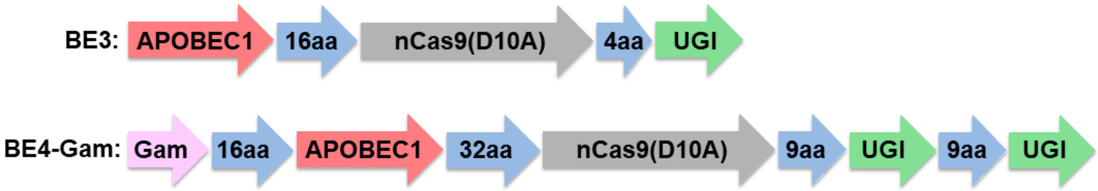

Supplementary Figure 6: **Architectures of BE3 and BE4-Gam system.** BE4-Gam system, with the Gam protein from bacteriophage Mu is fused to the N terminus, appending a second copy of UGI to the C terminus, and optimizing the length of linker.

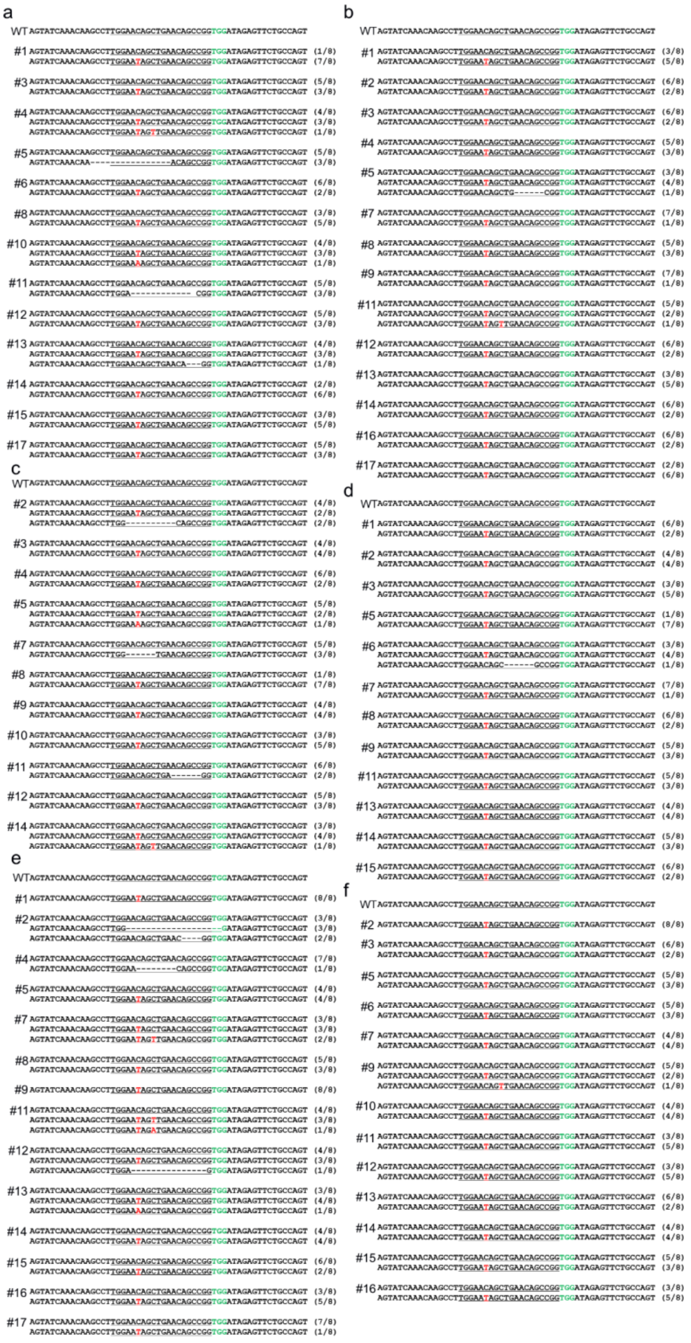

Supplementary Figure 7: **BE4-Gam system reduces indel frequencies, improves targeted mutation efficiency and product purity at *Dmd* locus in rabbit blastocysts.** (a, c and e): The genotypes of *Dmd* mutant blastocysts using BE3 system in Group 1 (a), 2 (c) and 3 (e), respectively. (b, d and f): The genotypes of *Dmd* mutant blastocysts using BE4-Gam system in Group 1 (b), 2 (d) and 3 (f), respectively. The number of clones for each sequence pattern is indicated. Target sequence (underlined), PAM region (green), and substituted nucleotide (red).

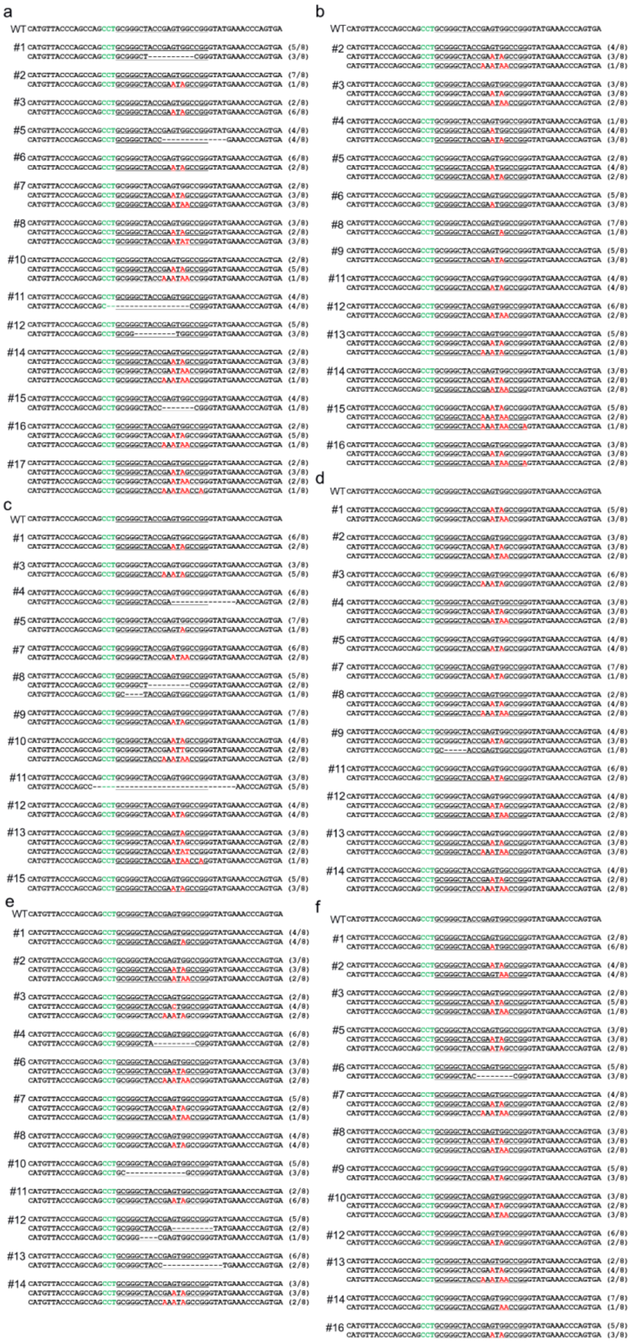

Supplementary Figure 8: **BE4-Gam system reduces indel frequencies, improves targeted mutation efficiency and product purity at *Tia1-2* locus in rabbit blastocysts.** (a, c and e): The genotypes of *Tia1-2* mutant blastocysts using BE3 system in Group 1 (a), 2 (c) and 3 (e), respectively. (b, d and f): The genotypes of *Tia1-2* mutant blastocysts using BE4-Gam system in Group 1 (b), 2 (d) and 3 (f), respectively. The number of clones for each sequence pattern is indicated. Target sequence (underlined), PAM region (green), and substituted nucleotide (red).

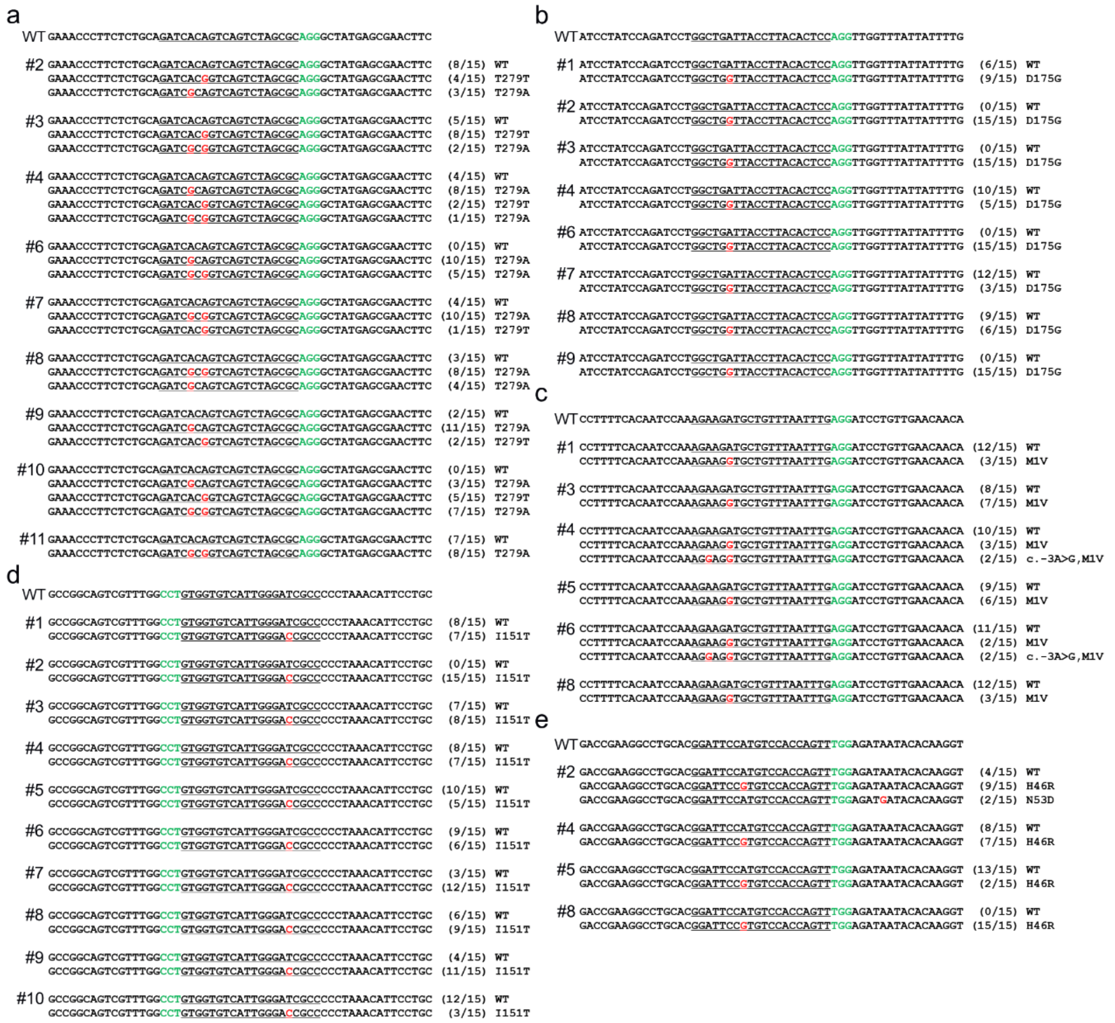

Supplementary Figure 9: **Targeted base editing in rabbit blastocysts using ABE7.10 system.** (a-e): PCR amplicons of *Dmd* (a), *Otc-1*(b), *Otc-2*(c), *Sod1-1*(d) and *Sod1-2* (e) loci from the genomic DNA of mutant embryos were subcloned into pGM-T vectors and sequenced, respectively. The number of clones for each sequence pattern is indicated. Target sequence (underlined), PAM region (green), and substituted nucleotide (red).

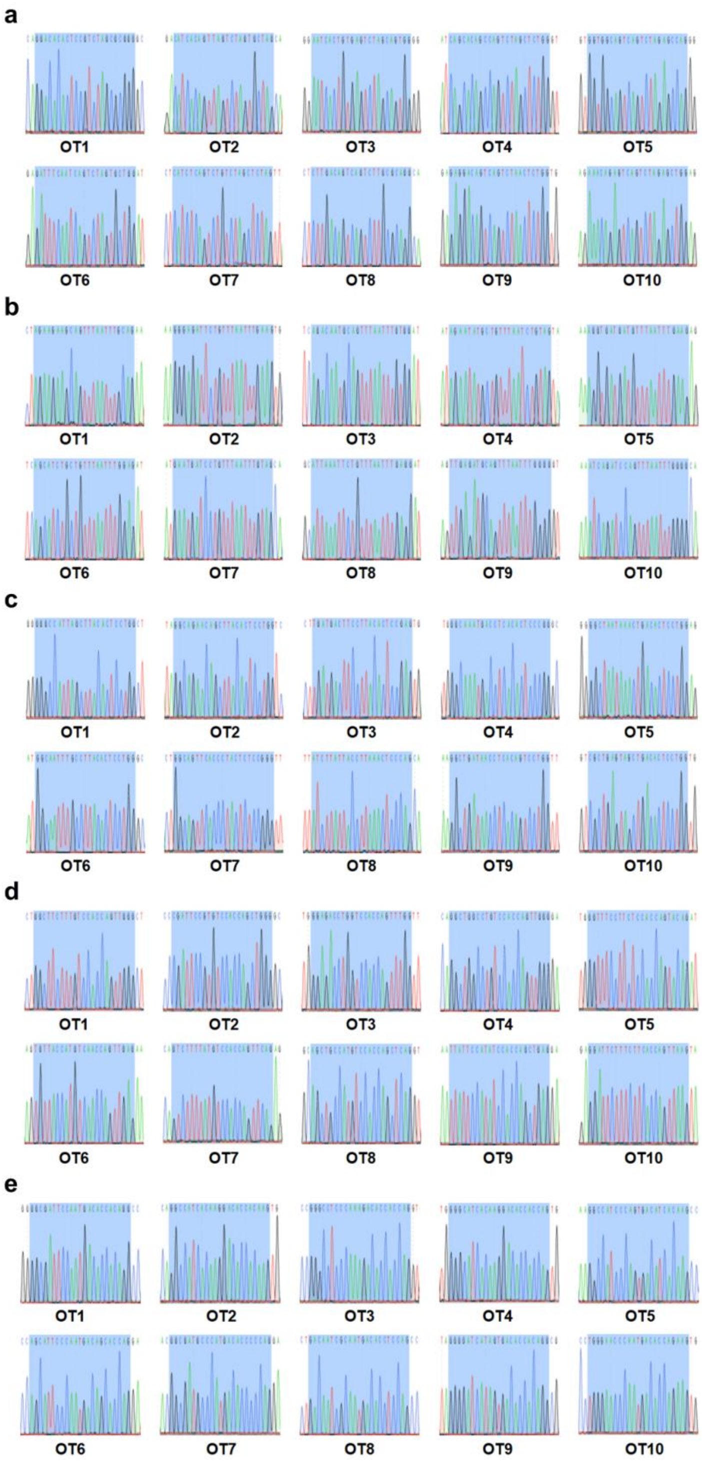

Supplementary Figure 10: **Off-target detection in the mutant rabbit blastocysts using ABE7.10 system.** (a-e): Chromatogram sequence analysis of ten POTS for sgRNA using PCR products in *Dmd* (a), *Otc-1* (b), *Otc-2* (c), *Sod1-1* (d) and *Sod1-2* (e) mutant rabbit blastocysts, respectively. 20 bp of the POTS and the PAM are represented in shadow.

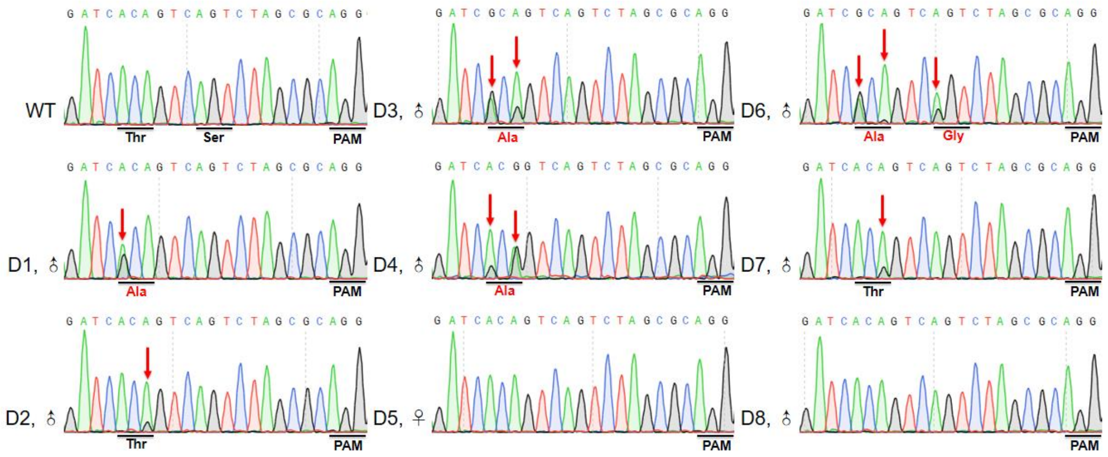

Supplementary Figure 11: **Sanger sequencing chromatograms of DNA from WT and *Dmd* Founder rabbits using ABE7.10 system.** Sanger sequencing chromatograms of genomic DNA from all *Dmd* Founder rabbits. The red arrow indicates the substituted nucleotide (A-G). The relevant codon identities at the target site are presented under the DNA sequence. The gender of each rabbit has been indicated. Male (♂), Female (♀).

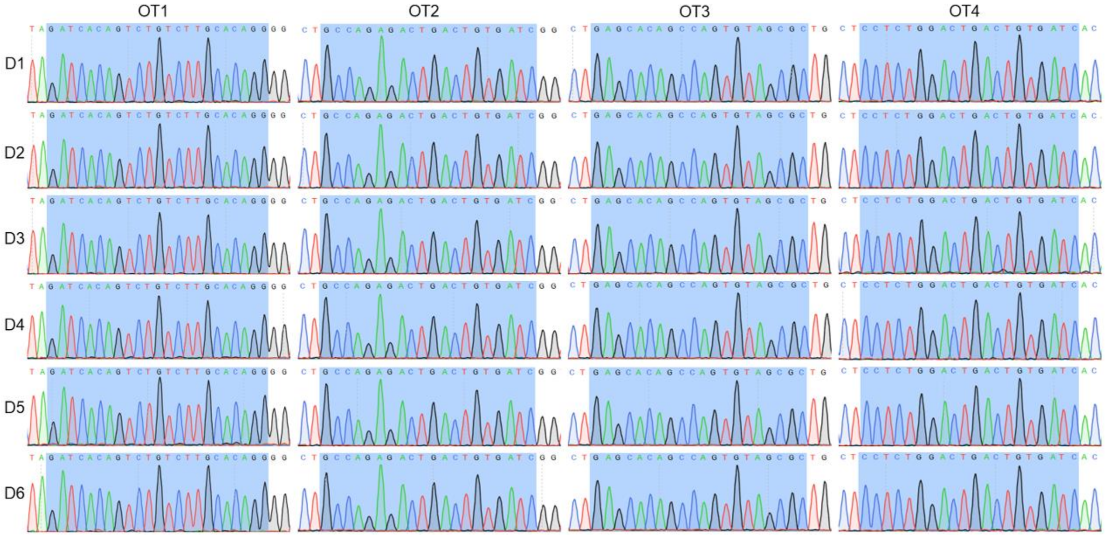

Supplementary Figure 12: **No off-target mutations were detectably induced at potential off-target sites in *Dmd* mutant rabbits.** Chromatogram sequence analysis of four potential off-target sites (POTS) for sgRNA using PCR products in *Dmd* mutant rabbits. 20 bp of the POTS and the PAM are represented as shaded.

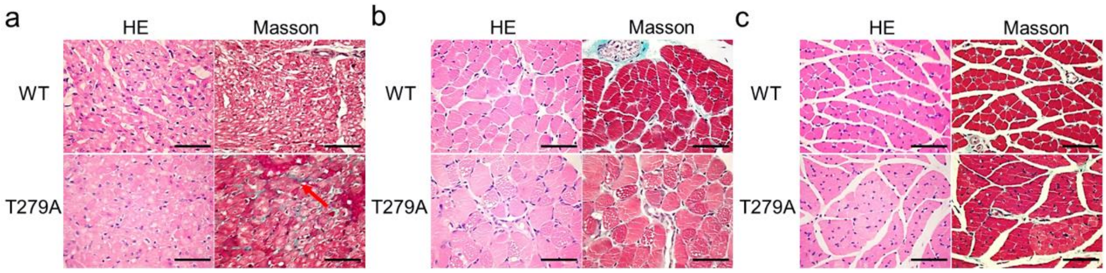

Supplementary Figure 13: **H&E-stained and Masson's trichrome-stained cross sections of cardiac muscle, diaphragm muscle and gastrocnemius from WT and *Dmd* T279A rabbits at 1 month of age.** (a): H&E-stained and Masson's trichrome-stained cross sections of cardiac muscle showed fibrosis (red arrows) in *Dmd* T279A rabbits. (b-c): There is no obvious pathological change in diaphragm muscle (b) and gastrocnemius (c) of *Dmd* T279A rabbits. Scale bars: 50 μm.

Supplementary Table 1: **Two strands of oligonucletotides were used to construct the pUC57-sgRNA vector.**

**System**	**sgRNA**	**Target site**	**PAM**	**Oligonucleotide1**	**Oligonucleotide2**
BE3 and BE4-Gam	*MSTN*	TGATCAGTACGACGTTCAGA	GGG	TAGGTGATCAGTACGACG TTCAGA	AAACTCTGAACGTCGTAC TGATCA
	Dmd	TGGAACAGCTGAACAGCCGG	TGG	TAGGTGGAACAGCTGAAC AGCCGG	AAACCCGGCTGTTCAGCT GTTCCA
	Tia1-1	CAGCCGCCTCCTGGCCAGAA	CGG	TAGGCAGCCGCCTCCTGG CCAGAA	AAACTTCTGGCCAGGAGG CGGCTG
	Tia1-2	CCGGCCACTCGGTAGCCCGC	AGG	TAGGCCGGCCACTCGGTA GCCCGC	AAACGCGGGCTACCGAGT GGCCGG
	Tyr	GCCTCAATTCCCCTTCACAG	GGG	TAGGCCTCAATTCCCCT TCACAG	AAACCTGTGAAGGGGAA TTGAGG
	Lmna	GTGGGCGGATCCATCTCCTC	TGG	TAGGTGGGCGGATCCAT CTCCTC	AAACGAGGAGATGGATC CGCCCA
ABE7.10	*Dmd*	GATCACAGTCAGTCTAGCGC	AGG	TAGGATCACAGTCAGTC TAGCGC	AAACGCGCTAGACTGAC TGTGAT
	Otc-1	GGCTGATTACCTTACACTCC	AGG	TAGGCTGATTACCTTA CACTCC	AAACGGAGTGTAAGGT AATCAG
	Otc-2	AGAAGATGCTGTTTAATTTG	AGG	TAGGAGAAGATGCTGTTT AATTTG	AAACCAAATTAAACAGCA TCTTCT
	Sod1-1	GGCGATCCCAATGACACCAC	AGG	TAGGCGATCCCAATGA CACCAC	AAACGTGGTGTCATTG GGATCG
	Sod1-2	GGATTCCATGTCCACCAGTT	TGG	TAGGATTCCATGTCCA CCAGTT	AAACAACTGGTGGACA TGGAAT
