# Supplementary material for: Highly efficient RNA-guided base editing in rabbit
Source: Nat Commun. 2018 Jul 13;9:2717. doi: 10.1038/s41467-018-05232-2 (PMC6045575; doi:10.1038/s41467-018-05232-2)
Supplement: Supplementary file 2 — Description of Additional Supplementary Files [file 41467_2018_5232_MOESM2_ESM.pdf]

Description of Additional Supplementary Files

File Name: Supplementary Data 1
Description: Primers used for PCR and sequencing in this study.

File Name: Supplementary Data 2
Description: The primers of potential off-target sites (POTS) used in this study at Mstn, Tyr, Lmna and Dmd loci.

File Name: Supplementary Data 3
Description: The primers of potential off-target sites (POTS) used in this study at Dmd, Otc-1, Otc-2, Sod1-1 and Sod1-2 loci.
